# Medical Journalism

**Published:** 1910-09-03

**Authors:** 


### MEDICAL JOURNALISM.

The death this year of a young medical man who had given up professional practice to join the staff of a medical contemporary draws attention to a bypath of medicine which of late years has become somewhat widened and more frequented. It is partly the growth of journalism in general which has brought this about, but more the rapid forward march of medicine itself; and things being so, medical journalism as a possible career perhaps calls for notice in the present issue of this journal, although it must be said at once that the opportunities are of the fewest. However, men and women really fitted for the work are probably less numerous still. Strange as the assertion may appear, far fewer of a year's entry of students have it in them to be first-class medical journalists than to be first-class consultants, the reason being, of course, that literary ability is not nearly so common as the broader, less specialised mental capacity called for in the ordinary pursuit of medicine. Of such ability the early signs are well known. A boy, less commonly a girl, will learn to read, if given the chance, at three or four years of age. He will be for ever found with a book, and his taste, at first omnivorous and undiscriminating, will with great quickness respond, and go on responding, to cultivation. After this stage, the characteristics to be looked for depend upon to which of two broad classes the aspirant belongs.

Readers of *Pendennis* will remember the contrast which Thackeray, with unconscious artistic truth, draws between the talents of his hero and Warrington. Pen can dash off a pretty little or funny little set of verses on a given pretty little or funny little subject; Warrington is a valued if anonymous worker upon the staff of the best reviews of the day. One has the creative faculty, the other the critical; and although, as happens to suit Thackeray's purpose in this case, the faculty in the former instance is exceedingly slight, and in the latter "constructive" and among the best of its kind, yet nevertheless they are things quite apart. The first, and only the first, is altogether impossible of acquirement. It may freely be granted that, as the French saying goes, "Il y a une foule de degrés." Douglas Jerrold's scorn may be allowed when, commenting on the poetaster's claim of kinship with Shakespeare, he instances contrasts strong enough merely in degree, such as those between the lion of the desert and a blind kitten, or a draught of Rhine wine and half a tumblerful of small beer, flat, and with dead flies in it. Nevertheless, writers without artistic imagination are generally considered, *quâ* writing, to belong to the inferior class. They are certainly less suited to the occupation under notice, for the humdrum reason that by comparison they lack the power of sustained production, of responding well to the inevitable demand for continuous literary output. The marks of this desirable "state of mind, conjoined to true reason, apt to make" (if Aristotle's words be not too sonorous to quote here) are evident enough. Among the youth's relatives will be actors or painters or musicians; at any rate (Mr. Henry James is a little nearer the proper tone), "a sketching grandmother . . . a building or versifying or collecting . . . ancestor." Curiously, but undoubtedly, he is further likely to have one parent a foreigner. And, to get completely back to our subject, he is likely as well, for the reason that artistic impulse shows itself early (if not ousted by a pushing individual who has been to a fashionable public school), to be found happily editing his hospital magazine.

Editing a hospital magazine is naturally good experience, although these journals are usually better from the literary than from the journalistic point of view, because good journalism regularly pursued means very hard work, and the medical student has already as much work provided for him as the youthful mind craves for. But the habit of restraint inculcated by responsibility for the written word, and the need for studying readers' tastes, can be learnt very well in this school. A little experience in conducting the correspondence columns will soon impress on him the vast difference between the itch for writing and the capacity for it. Friends and acquaintances and some little reputation may be made, acquisitions all useful in obtaining a set in the right direction—that direction being, of course, towards certain easily discernible positions.

But, be it repeated, these positions are exacting. We have supposed the possession of natural aptitude; that is, of wide reading; of the literary memory that retains what is read just as well as, in fact better than, what one hears; of some infusion of Macaulay's faculty of reading as fast as ordinary people skim; of the born writer's interest in words, in the arrangement of sentences, in the whole art and craft of literary expression; we have supposed all these and a good deal more. Besides it all is needed a liberal education. Medical training is but engrafted on acquaintance with the classics, with philosophy, science, and political economy. To be able to read the four Congress languages and to speak French and German are almost essential. A knowledge of politics and education in their relations to medicine will be acquired, but in addition to the above general equipment the aspirant will do well to make himself master of some subject, say, psychology, or the history of medicine, which happens to have intrinsic attraction for him.

As to the agreeables and disagreeables of medical journalism, there is, of course, much hack-work to be done; more informative and less imaginative writing is called for than in lay periodicals. Nevertheless, medicine is very rich in its wide and increasing relations with other departments of human thought and activity. That this is so, and that the feature can be well reflected in a medical press, any observer can convince himself by comparing, say, the *British Medical Journal* with the *Law Times*. And although the influence to be exerted on opinion is not so great as in general journalism, yet it is real enough too. Finally, there is the interest of meeting in colleagues those who are also fond of the contemplative life, of appreciating their talent, of learning from them and discussing with them. These, and the exhausting joys of conscientious composition, are some things to put against a long apprenticeship and an uncertain future.

